# A new SLA-aware method for discovering the cloud services using an improved nature-inspired optimization algorithm

**DOI:** 10.7717/peerj-cs.539

**Published:** 2021-05-10

**Authors:** Arash Heidari, Nima Jafari Navimipour

**Affiliations:** 1 Department of Computer Engineering, Tabriz Branch, Islamic Azad University, Tabriz, Iran; 2 Future Technology Research Center, National Yunlin University of Science and Technology, Douliu, Taiwan

**Keywords:** Cloud computing, Resource, Inverted ant colony, Service, Discovery, SLA

## Abstract

Cloud computing is one of the most important computing patterns that use a pay-as-you-go manner to process data and execute applications. Therefore, numerous enterprises are migrating their applications to cloud environments. Not only do intensive applications deal with enormous quantities of data, but they also demonstrate compute-intensive properties very frequently. The dynamicity, coupled with the ambiguity between marketed resources and resource requirement queries from users, remains important issues that hamper efficient discovery in a cloud environment. Cloud service discovery becomes a complex problem because of the increase in network size and complexity. Complexity and network size keep increasing dynamically, making it a complex NP-hard problem that requires effective service discovery approaches. One of the most famous cloud service discovery methods is the Ant Colony Optimization (ACO) algorithm; however, it suffers from a load balancing problem among the discovered nodes. If the workload balance is inefficient, it limits the use of resources. This paper solved this problem by applying an Inverted Ant Colony Optimization (IACO) algorithm for load-aware service discovery in cloud computing. The IACO considers the pheromones’ repulsion instead of attraction. We design a model for service discovery in the cloud environment to overcome the traditional shortcomings. Numerical results demonstrate that the proposed mechanism can obtain an efficient service discovery method. The algorithm is simulated using a CloudSim simulator, and the result shows better performance. Reducing energy consumption, mitigate response time, and better Service Level Agreement (SLA) violation in the cloud environments are the advantages of the proposed method.

## Introduction

Cloud computing consolidates itself for the next steps of increasing the number of distributed services (Sheikholeslami & Navimipour, 2017; Sheikholeslami & Jafari Navimipour, 2018). Therefore, the services based on cloud computing have a rapid growth in recent years (Chiregi & Navimipour, 2017; Al-Sayed, Hassan & Omara, 2020). Some resources and services, such as storage, processing, network bandwidth, and Virtual Machine (VM), are included in the cloud (Dordaie & Navimipour, 2017). In this environment, the capabilities are also dynamically enhanced without dedicating new infrastructure, training new workers, or licensing new software (Milani & Navimipour, 2016). Using cloud computing, industry and academia organizations must lease their services (Thai, Varghese & Barker, 2018; Heidari et al., 2020). Virtualized resources, scalable data storage, parallel processing, and security are among the benefits of cloud computing (Mahmud, Iqbal & Doctor, 2016; Ravi et al., 2017). The high potential of cloud computing provides tremendous benefits to the industry and the community (Ali, Khan & Vasilakos, 2015; Asghari & Navimipour, 2018).

Nowadays, cloud services' development offers a wide source of information that causes many problems in this subject (Jafari Kaleibar & Abbaspour, 2020). Without thinking carefully about some circumstances, such as functional, non-functional, and budgetary limitations, it is not probable to discover the numerous cloud providers (Kapil et al., 2020). Therefore, efficient service discovery in the cloud is a significant challenge and an essential part of any distributed system (Srirama, Paniagua & Liivi, 2013; Navimipour et al., 2014; Egbe et al., 2016). However, cloud computing managers can automatically or semi-automatically detect services or service information (Afify et al., 2014; Zhang et al., 2019).

While the complexity and network size grow dynamically, the service discovery becomes an NP-hard problem in which efficient algorithms are required to discover optimum resources (Ezugwu & Adewumi, 2017; Md, Varadarajan & Mandal, 2019). For this reason, service discovery is one of the indispensable challenges in the cloud, which finds suitable resources based on the requested job (Navimipour et al., 2014; Rajendran & Swamynathan, 2019). Moreover, service discovery becomes more important as distributed systems grow and their pool of resources becomes more variable (Tianxing et al., 2020). Finding effective and efficient ways to discover services in these systems is necessary to increase the number of users and services in cloud computing systems (Ali et al., 2020). Some factors, including the huge number of resources, distributed ownership, heterogeneity of services, service failure, reliability, scalability, and service evolution, make the service discovery problem difficult. These factors are vital criteria for designing a suitable service discovery mechanism (Navimipour et al., 2014).

On the other hand, load balancing is one of the main methods by which energy optimization is accomplished. Researchers have placed forward resource scheduling with load balancing algorithms under different circumstances. Since very few papers are aimed at load-balanced discovering of the services among cloud servers to avoid creating bottlenecks on servers, therefore, in this paper, load balancing is a side aim of the service discovery. Each service discovery requires the necessary collection of available services that include various features and meet the required Quality of Service (QoS) measures. The desired QoS values are maintained as a contract between the customer and the service provider in a formal document called SLA and imposes penalties in case of breach of the QoS specifications by providers. In this paper, the authors describe the SLA variations as the number of unmet requests to the number of requests that have been met. Therefore, in this paper, a Cloud Service Discovery method based on an Inverted Ant Colony Optimization algorithm (CSD_IACO) is proposed to discover relevant services and solve the weaknesses of traditional service discovery mechanisms in the cloud environment by considering the SLA. In this method, the pheromones have repulsion instead of attraction. This repulsion causes avoiding overhead on the same route to find proper services based on the user requirements. Similar to the ACO algorithm, tasks (jobs/cloudlets) are given to several ants. The modified pheromone has a major impact on improving load balancing between cloud servers in each iteration. After applying modified pheromone, ants try to select new paths to cross and obey the requester’s specifications. In a nutshell, the broker is responsible for distributing ants (each ant includes a task) to the servers in the proposed method. By starting a request, the ants moving into the cloud and looking for various services such as a computational resource. So, since the traveled paths are stored in the path-matrix, it avoids choosing the same paths by the same ant. In the final round, the best services, which have the minimum processing time in the previous step, are selected and allocated to the user. Briefly, the significant contributions of the paper are:

- Establishing load balancing between cloud servers using the CSD_IACO technique.
- Proposing a new service discovery mechanism named CSD_IACO to optimize response time, SLA differential, and total energy consumption.
- Comparing the CSD_IACO technique with three state-of-the-arts and declaration the superiority of it.

The following categorization will be discussed in the rest of this paper. “Related Work” and “Proposed Method” refer to discuss related work and the proposed method. The result of the simulation is presented in “Results”. In the end, conclusions and future works are presented in “Conclusion, Limitations, and Future Work”.

## Related Work

Service discovery in cloud computing involves locating the required services according to service descriptions (Hajlaoui et al., 2017). Effective and efficient service discovery is essential in cloud environments. However, designing an efficient service discovery method incorporates some issues, namely response time, robustness, cost, energy consumption, and scalability (Athwani & Vidyarthi, 2015). Centralized, decentralized, and hybrid are the three main categories of service/resource discovery mechanisms in cloud environments. Moreover, in this classification, P2P and agent-based mechanisms are considered as a part of the decentralized mechanism. This section describes six mechanisms of service/resource discovery in cloud environments and elaborates their basic properties.

A Soft Set Symbiotic Organisms Search (SSSOS) algorithm was presented by Ezugwu & Adewumi (2017) for optimizing a service selection method in the cloud. The method includes the principle of reducing the characteristics of soft objects and the coexistence of organisms in an ecosystem. These two selection methods are used to search for the perfect solving method that attains the subscribers’ needs. The soft collection choosing method accelerates the search procedure by building a service cluster-info table using frequency information of single service parameter properties and a weight method that categorizes the services depending on the belonging property values. The SSSOS is effective, and the obtained results announce the algorithm’s promising potential for cloud service discovery. The algorithm minimizes the service selection time and has high search precision. However, it suffers from high complexity and low scalability.

Based on an IACO algorithm, Asghari & Navimipour (2019) proposed a resource discovery technique in P2P networks. In this method, a query of the requester was sent to the neighbor peers in the first segment, the neighbor peers tried to respond to the query, and the answer message was returned to the requester. The optimal peer that contained the best value of probability was chosen in the third phase. Finally, the quantity and specifications of pheromones have been revised, allowing the suggested technique to boost peer load balancing. The experiments focused on the number of peers, the number of target resources, the resources available to each peer, the number of peers in the neighborhood, the graph’s completeness, and the number of ants. The results showed that, in terms of load balancing, waiting time, and resource usage, the IACO was superior to the ACO algorithm. This method is based on the P2P networks, and the SLA parameter is not investigated.

Also, Ananthi et al. (2020) presented a paradigm for the secure and proper discovery of outsourced services on the cloud platform. Their semantic framework of discovery is fuzzy-based, ensuring that the relevant results are returned consistently and supporting the total user experience. Besides, the paradigm overcomes overhead processing by reducing the time taken to create index files. To delete the sorting and some other errors in the query strings, semantic matching of keywords is performed, which involves substituting terms to obtain sufficient data without any difficulty. The relevance score is defined, and grade-based data retrieval is carried out based on highly relevant information. As a result, the user quickly and accurately discovers the requested service in less response time. The proposed mechanism, besides, has high complexity and low availability. It still improves the efficiency of searching and supports multi-attribute queries. Also, SLA was not considered in this study.

Furthermore, an efficient multi-agent reference architecture and cloud ontology for cloud services discovery was proposed by Abbas et al. (2020). The proposed architecture addresses the vendor’s locked-in problem to some extent and mitigates the problems of portability and interoperability in the cloud environment. Several intelligent agents exist in the multi-agent scheme, such as a directory agent, a discovery agent, a ranking agent, a user agent, etc. Cloud ontology for cloud-based knowledge acquisition is another key component of this architecture. For creating and implementing a reference architecture, a systematic methodology has been presented. It is used to build smart agents and to ensure multi-agent system interoperability. The outcome showed that the reference architecture in terms of search efficiency, response time, and execution time is better than the current methods.

Moorthy & Pabitha (2020) proposed a sine coined clustering-based method combined with a cloud-based service discovery mechanism. A metaheuristic algorithm called sine cosine optimization is used to evaluate the maximum service discovery. The service distribution search space has been limited through the use of clustering. In terms of inter-cluster similarity, their approach contrasts with the effectiveness of K-Means, inter-cluster similarity, and service discovery. The results showed that in the identical cluster, the system performs low inter-cluster similarity, maximum intra-cluster similarity, and high service similarity, making the optimum clustering relative to K-Means. This mechanism is effective for cluster-based distributed environments, but for heterogeneous, large-scale, and dynamically interoperable infrastructures like inter-clouds, it is questionable. This system has a high cost and low security. Nevertheless, it increases search performance and provides a high tolerance for failures. Besides, SLA was not considered in this paper.

Finally, a framework called Praxi was developed by Byrne et al. (2020) for service discovery in cloud environments. Praxi incorporates the powerful points of previously proposed learning-based approaches to provide a fully automated exploration methodology that does not involve rigorous learning to minimize and anticipate characteristics, thereby alleviating their weaknesses. Besides, their strategy makes incremental learning easier and faster. The results showed that their system could support accurate discovery, reduced runtime, and lower overhead storage. The mechanism suffers from high complexity and long processing time for queries. Moreover, its weak points are inadequate security and scalability. Also, SLA was not considered in this paper.

## Proposed Method

One of the problems in providing cloud services is the use of idle machines for data processing. Due to high energy consumption and produced carbon dioxide by these devices, cloud providers reduce energy consumption and response time of required services. Also, the proper allocation of VMs and exact service discovery applications allows them to enhance QoS in terms of cost, performance, efficiency, response time, SLA differential, etc.

The reduction of energy consumption and optimizing the resource management process are remarkable problems in cloud systems. Hence, this paper addresses this issue. For this purpose, we will first examine the ACO algorithm to solve this problem. Based on this algorithm, the ants represent a task that moves through the machines (servers/VMs), and the best possible machine can perform the task with the least energy. In this paper’s considered algorithm, we have solved the original algorithm’s weakness using the CSD_IACO algorithm. In this method, similar to the ACO algorithm, tasks are given to several ants. But, in this method, the pheromones are based on the amount of consumed energy per machine, and the algorithm’s mathematical relation will be decreased. Also, the pheromones will be stored inside the nodes. Yet, the pheromones will not exist along the routes. After reviewing these two algorithms, a graphical example of this system will be investigated.

### Cloud model

The cloud model is based on the Internet (Jamali et al., 2020). Its usage is very easy and has access to computing resources such as servers, storage, applications, and services. Cloud computing is one of the distributed and parallel systems that includes a set of VMs and computers which are connected. These computers are dynamically delivered and are realized as one or more integrated computing services based on the SLA. The agreements are being negotiated between service providers and consumers (De Carvalho et al., 2017).

**Figure 1:**
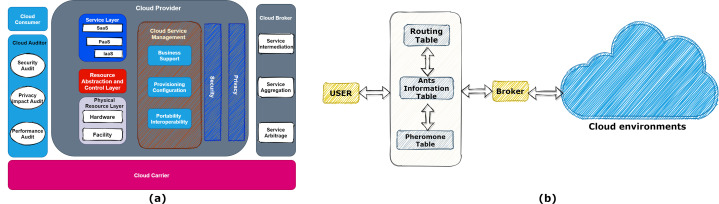
(A) Different layers of the cloud computing model. (B) Discovery model.

Figure 1A shows the different layers of the cloud. In each cloud system, we can extend the data center’s existence to model infrastructure services in the simulator. A simulator such as CloudSim is an application, library, or framework designed to provide and test a realistic imitation of cloud infrastructures and services used for research aims. The data center manages a large number of entities, such as hosts and VMs. Finding the appropriate host for every task is an issue of great importance. For this reason, the service discovery system finds the best service with the most matching on user demands and then offers them to the broker. A cloud broker is a unit that manages the use, efficiency, and delivery of cloud services and negotiates relationships between cloud providers and customers. However, the service discovery system tries to offer the broker’s best available services as a software system. With this description, the correct design of this unit can be very promising.

The proposed mechanism model that includes all the user interface, cloud environments, ants’ management, and broker is shown in Fig. 1B. Also, the requested services (tasks) are stored in a matrix. The broker splits the tasks (applications that needed to be computed/processed in the cloud) between hosts and VMs.

### ACO algorithm

The service discovery should be able to locate services for cloud users when they submit their queries. By requesting a user, several ants (tasks/jobs) search for the required services simultaneously. However, real ants and the searched sources are the basis for proposing an ACO algorithm in which the nodes are machines, and each ant has a job to find the best machine. Each ant produces pheromones on its path. The first node is randomly selected, but for other nodes, the probability of selection is calculated. However, the node that is more likely to be chosen is selected. The Eq. (1) calculates the probability of selecting any path by an ant (Manogaran et al., 2019).

(1) $$p_{i,j}^{k}=\frac{{\left[\tau_{i,j}\right]}^{\alpha }{\left[n_{i,j}\right]}^{\beta }}{\sum_{l=j_{i}}{\left[\tau_{i,l}\right]}^{\alpha }{\left[n_{i,l}\right]}^{\beta }}$$

where $\tau_{i,j}$ is the amount of pheromones in the path i to j and $n_{i,j}$ is the reverse of the distance between the two nodes i and j and *α* and *β* are the control parameters. In the proposed method, the distance between two nodes i and j is the consumed energy for moving in the machine i to j, which is also true in the CSD_IACO.

The ants should return to their nests as soon as they get food (appropriate service concerning user demands) drop pheromone on trails (the path between machines/hosts/VMs in the cloud). The pheromone trail amount can either increase as ants deposit pheromone or decrease due to pheromone evaporation as calculated based on Eq. (2) (Dorigo, Birattari & Stutzle, 2006). Where (1−ω) is a pheromone declining rate and $\Delta \tau_{i,j}$ is the pheromone quantity present in the route (i,j), most recently updated by the $m_{th}$ ant.

(2) $$\tau_{i,j}=\left(1-\omega \right)*\tau_{i,j}+\Sigma_{r=1\;}\Delta {\tau^{r}}_{i,j}$$

Other ants follow the same trail with a high amount of pheromone. There are usually two approaches to update the pheromone trails. (1) That first one is to select the iteration-best or best-so-far solutions to update the pheromone matrices concerning each objective. (2) A second approach is to gathering and store the non-dominated solutions in an external set. Only the answers in the non-dominated set are permissible to update the pheromones. Finally, they will return home after finding food and support the pheromone on the trail. The local decisions that ants make are based on their observations and the local environment information. Other ants use indirect forms of communication instead of direct communication with each other. The term ‘stigmergy’ is used to call indirect communication between ants. The pheromones evaporate the key over time. More pheromone will be evaporated when returning an ant to its nest takes a long time. The intersection is the place where each ant should choose one of the branches. The ants chose a short branch to return home faster than those chosen a long branch (Deng, Wang & Ciura, 2009).

**Figure 2:**
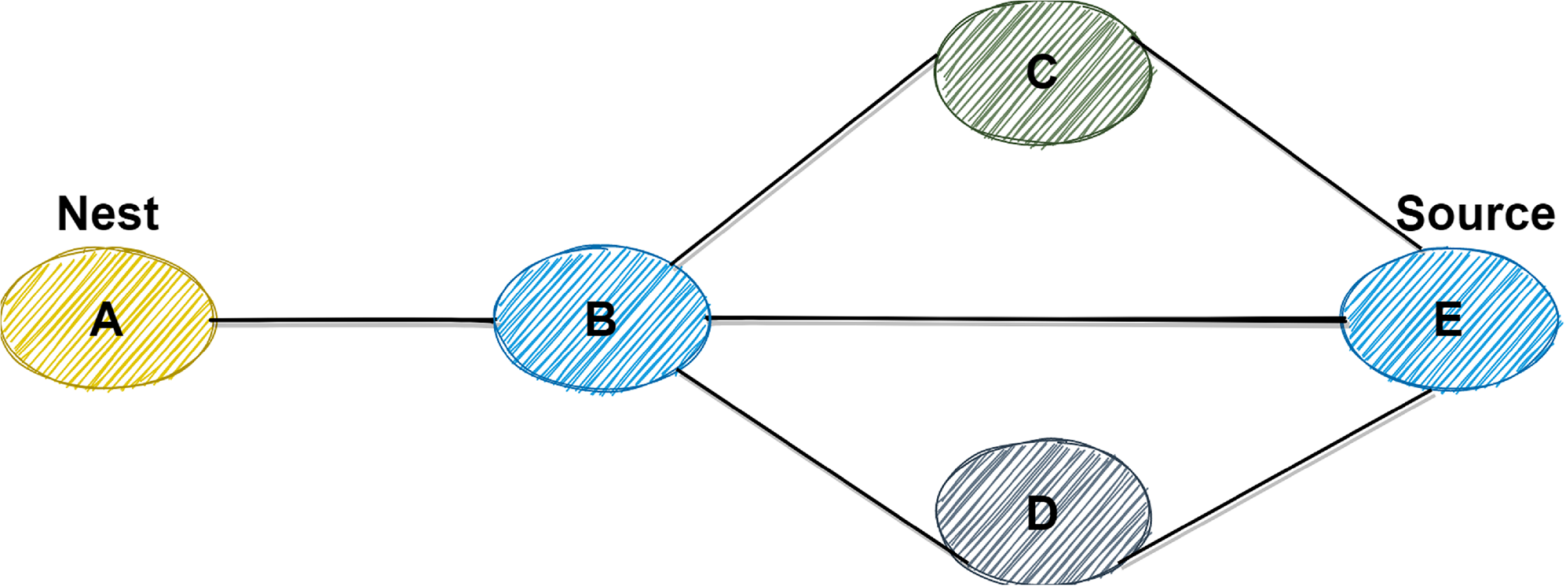
Example of ACO in terms of path selection.

Thus, the short paths have high pheromone density. The branches are selected by other ants that have pheromone densities. Eventually, all the ants select the shortest path to get food or sources. A simple example of the above scenario is presented in Fig. 2. Initially, ants are faced up with three paths: *A→B→C→E, A→B→E*, and *A→B→D→E*. After the initial step, most ants will take the shortest path, which is *A→B→E*. In the following, a description of the CSD_IACO is the main topic of this article.

### CSD_IACO method

The CSD_IACO has more significant differences in each ant’s behavior rather than ACO. There are three main differences in the ACO and CSD_IACO method as follows: Firstly, the pheromones which ants produce in the ACO method only have the function of attraction. In contrast, this pheromone acts as a repulsion in the CSD_IACO to reduce the pressure on a path or a node. Secondly, in the ACO, the nodes do not act as a parameter in the algorithm, and only the paths and pheromones presented on these paths are used as important parameters. While in the CSD_IACO method, nodes act as an influencing parameter; they store the number of pheromones. If these pheromones are more than a specific value, the way to enter the other ants through this node is closed. However, this action aims to reduce the pressure, which is directly related to the pheromone value. Thirdly, The ACO is not considered a parameter as the maximum capacity on a path. But, in CSD_IACO, the pheromone’s evaporation is based on the route’s length and the maximum route capacity.

These three differences will make important changes in the CSD_IACO method. According to this method, we can calculate the energy consumption, response time, and SLA differences. Each ant in this system represents a task, and each machine represents a source or service. Each ant moves between machines during its life cycle and tries to find the best service to do its task. Based on the following equation, the number of pheromones in each node will be calculated based on (3) and (4) (Dias et al., 2014). SLA differences will also be determined using (5) (Van, Tran & Menaud, 2009).

(3) $$minTT_{edge}=\frac{edge\;length}{edgeMaxAllowedSpeed}\;\;\;\;\;\;\;\;\;\;\;\;\;\;\;\;\;\;\;\;\;\;\;\;\;\;\;\;\;\;\;\;\;\;\;$$

(4) $$pher_{edge}=\{\begin{array}{c} pher_{edge}+DEP_{pher}\\ Pher_{edge}-minTT_{edge}\times DEP_{PHER} \end{array}$$

(5) SLA=Totalrecivedrequests−Requestsmade

In Eqs. (3) and (4), the edgeMaxAllowedSpeed represents the maximum energy, and the amount of the edge length is the amount of energy produced by the task in each machine. Whatever the amount of Eq. (3) is less, the repulsion rate is lower. As a result, according to Eq. (4), the amount of stored pheromone per node is decreased very slightly. Based on the CSD_IACO, if more pheromone is stored in each machine, there is more chance to select the ultimate machine by an ant. Thus, every task that an ant carries will achieve the best possible service. In fact, in the inversion of the ACO algorithm, the pheromones have repulsion instead of attraction. It consists of having the pheromones causing repulsion instead of attraction. The result of converting attraction to repulsion is to avoid dense paths that these results are load balancing and load distribution. The pseudocode of the CSD_IACO algorithm is demonstrated in Algorithm 1.

**Algorithm 1:** CSD_IACO.

**Begin**
Input();
Initial a max_ttl; Ant_count; Variable declaration;
**while** (step < max_ttl)
**{**
**For** (int i=0; i<ant_count; i++)
**{**
$MIN\_TTL=\frac{\cos t_{i}}{\text{edge\_max\_speed}}$
pher_edge[i][ant[i][1]]=pher_edge[i][ant[i][1]]+dep_phr;
pher_edge[i][ant[i][1]]=pher_edge[i][ant[i][1]]-MIN_TTL*dep_phr;
**}**
**for**(int i=0;i<ant_count; i++)
next step(i);
step++;
**end while**
**}**
Best ant pheromone => detect maximum pheromone in all machine
Post process results and visualization;
**end**

### Flowchart of CSD_IACO

In summary, the steps of the algorithm are as follows:

1. Initialization
2. Move between machines (based on determined TTL)
3. Choose the best pheromone
4. Create a VMs cloudlet matrix
5. Calculate the final energy consumption

**Figure 3:**
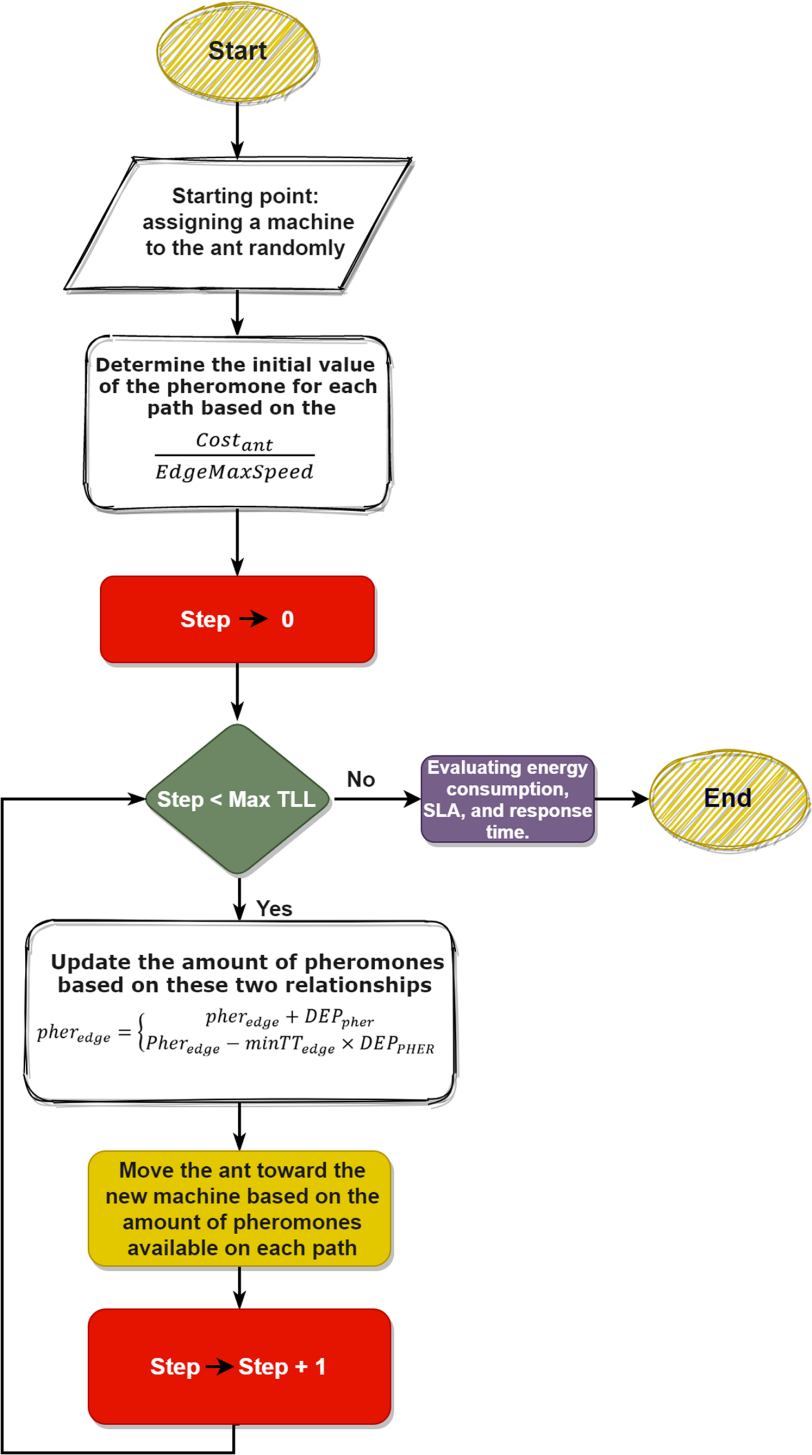
Flowchart of the proposed method.

Also, the flowchart of the proposed method is depicted in Fig. 3. In the CSD_IACO process, each ant is assigned an ID, and users send queries after initializing the system parameters. Each ant represents a task, so the ants start moving between the nodes, the first move, and the first node is selected randomly, and if this node is already chosen, the ant chooses another node. It should be noted that the number of machines meeting each ant depends entirely on the given TTL value. The deposit initial pheromone value will be assigned after reaching the node. The machine cost is computed after the ant enters the node. The Next_Step function will specify the next path of an ant after calculating the cost. The ant randomly selects another node in this function from the paths it has not yet selected and the nodes it has not reached. For all ants, this process will continue until TTL is completed. However, the nodes with the highest amount of pheromones will be selected after all the ants have completed their steps (based on the system’s initially specified values). After these steps have been completed, a new matrix called Cloudlet_VM will be created to fit all the acceptable nodes for all ants. Finally, based on Cloudlet_VM, the system calculates the final cost and prints the final values, i.e., the response time, the amount of energy consumed, and the SLA.

### Example

In this section, a simple example is examined to better clarifying and understanding the proposed method. The system consists of six tasks and six VMs. Each task has three specific properties: length-task, filesize-task, and output size-task. Initially, each task is randomly assigned to a machine by the broker. Then, based on the amount of energy that an ant uses in each machine, the pheromones’ values are determined. Afterward, in the next step, using the number of pheromones, the next machine is selected, and the ant is transmitted to it. In the end, the traversed route by each ant based on the amount of consumed energy is investigated. Finally, a machine that consumes less energy in the ant's movement path is used to perform the task carried by an ant. Based on the simple example, the system has six machines that must process six dedicated tasks and perform these processes with the least amount of energy.

**Figure 4:**
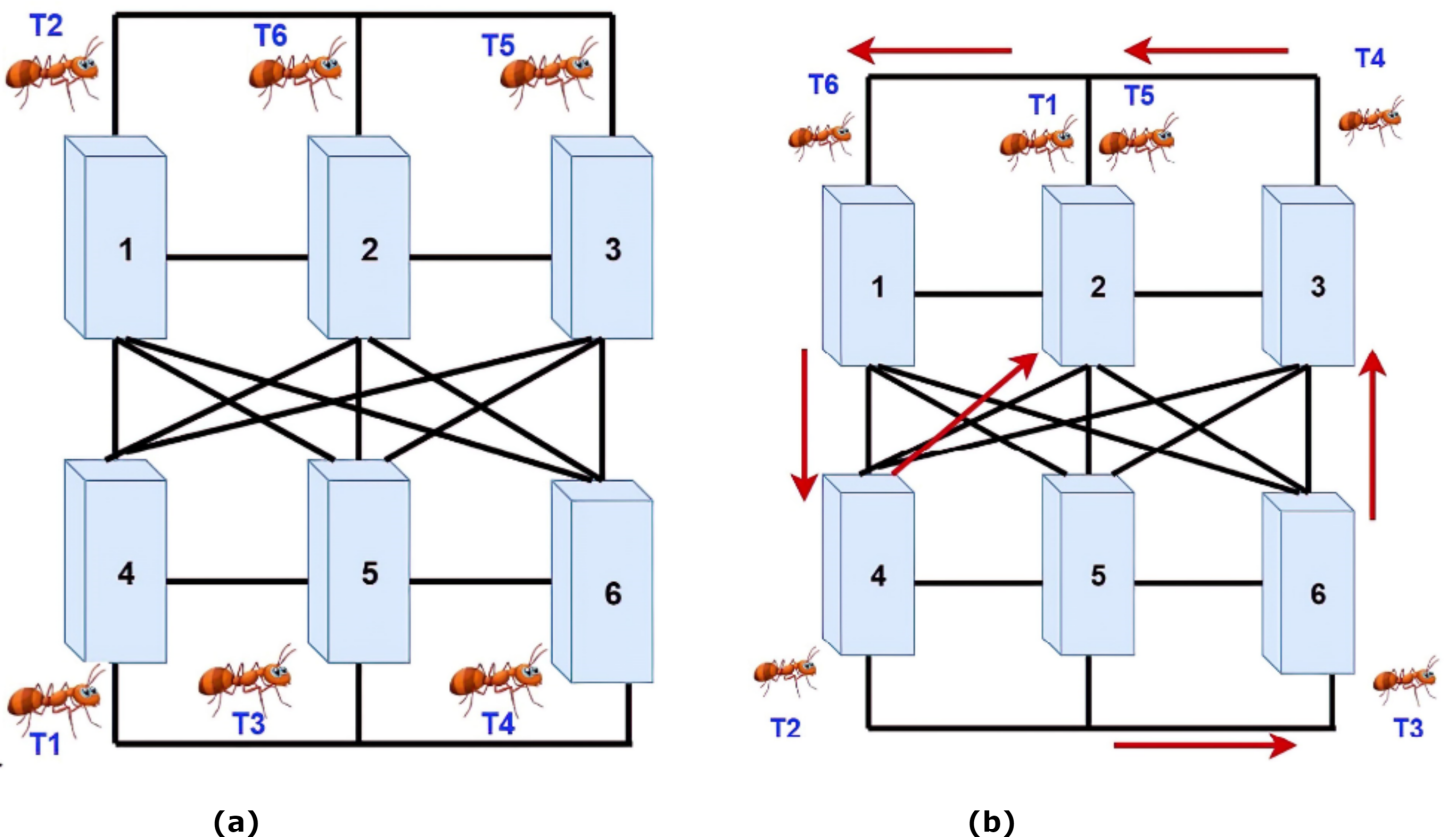
(A) Assigning ants to VMs. (B) Moving the ants to machines with more pheromones.

Firstly, six ants are randomly assigned to each VM as the starting point by the broker. Fig. 4A shows the assignment of ants to VMs. Secondly, based on the machines' assignment of tasks, the amount of consumed energy for each task in the machine is calculated. However, based on two Eqs. (3) and (4), the amount of stored pheromone is calculated per machine. Then the ants begin to move. Therefore, based on the pheromones in the ants’ neighborhoods, they choose one of the machines. In this system, the priority of choices is for heavy processes. Also, if the machine can do more than one job, two ants can move at the same time to a machine. As shown in Fig. 4B, both ants T1 and T5 are moved towards machine No. 2. Moreover, the ants move between the machines until it reaches its maximum steps. When the ants arrived at their last move, according to the priority of the pheromones’ size, the tasks are allocated to the machines. Therefore, it is trying to get every machine up to one task at a time.

## Results

The proposed method is analyzed and examined in this section to validate the behavior of the proposed method. The method is simulated in NetBeans and CloudSim. We compare the CSD_IACO to the following three schemes. (1) ACO-inspired resource discovery method named ACOD (Deng, Wang & Ciura, 2009) (2) Soft sets based symbiotic organisms search algorithm for resource discovery in cloud computing called SSSD (Ezugwu & Adewumi, 2017) (3) FIPA-based architecture for the discovery of appropriate cloud service using cloud ontology named FIPA-CD (Abbas et al., 2020).

### Simulation environments

The proposed algorithm’s evaluation to test the energy consumption, response time, and SLA differential are performed in this section. In this paper, the system under consideration is represented as a data center containing heterogeneous nodes. Each node i has the following characteristics: (1) processing speed, which is measured with MIPS (Million Instructions Per Second), (2) network bandwidth, and (3) memory capacity. The tasks are given to the VMs, which have three attributes: processing power, memory capacity, and network bandwidth.

The ability to describe a simulation with a high degree of configuration and flexibility is the most important simulator feature. So, a simulator, especially in the case of modeling, depends on a large number of parameters, and most of the time the values of those parameters must be assumed. Therefore, entering and changing the parameters in the simulation should be simple. CloudSim is a Java-based simulator and is used for cloud simulation. It supports the behavior of system components modeling, such as data centers, VMs, and resource planning policies. However, we define components such as the data center, broker, and cloud information service as an entity in the CloudSim. Also, CloudSim suggests the underneath aspect: (1) backing for designing and simulation of largescale cloud environments, containing data centers, hosts, and so on; (2) a self-contained platform for modeling clouds such as private cloud or public cloud, create agents and brokers, provisioning, and allocation policies; (3) support for simulation of network linkage between the simulated system elements; and (4) easiness for simulation of federated cloud environment that inter-networks resources from both private and public domains, a trait critical for research studies relevant to cloud-bursts and automatic application scaling. Several of the unique aspects of CloudSim are (1) availability of a virtualization engine that aids in the development and handling of innumerable, autonomous, and co-hosted virtualized services on a data center node and (2) flexibility to substitution among time-shared and space-shared allocation of processing cores to virtualized services. These compelling aspects of CloudSim tend to speed up the expansion of new application provisioning algorithms for cloud computing (Calheiros et al., 2011).

#### Datacenter and host class

We can extend the data center model, such as infrastructure services in the simulator. The data center manages many hosts by the policy of assigning VMs defined by the cloud service provider. These hosts are assigned to VMs. In the simulator, the VM policy represents VM control policies related to its lifetime, such as providing hosts for VMs, creating and removing them, plus migrating these VMs. Similarly, one or more functional services can be assigned to the VM, which is the same preparation for applications in the cloud environment. A component in the CloudSim can be a class (abstract or complete) or a set of classes that represents a model (data center or host). A small amount of energy consumed is spent on cloud computing centers, and its largest amount is spent on other processes such as data center coolers, lighting, and construction. However, one of the important items for reducing energy consumption at the computational level is the proper allocation of tasks to VMs, which can be achieved through the right allocation of tasks, preventing unmanaged migration, reducing energy consumption, and increasing efficiency. One data center is defined in our work. The data center is used to define the physical devices that are used. Therefore, these devices can be homogeneous or heterogeneous. The hardware of the system is explained in this section. Also, in this class, the capacity of memory, processing power, and the number of CPUs are defined. Exemplary host class and VM characteristics are also defined in Table 1.

**Table 1:** Host class and VM characteristics.

Specification of the host class		Specification of VM
CPU Speed	1,000, 2,000, 3,000 MIPS	250, 500, 750, 1,000 MIPS
RAM	10,000 MB	128 MB
BW	100,000 Mbit/s	200 Mbit/s
HARD	1,000,000 GB	250 GB

In the host class, we have modeled physical resources, such as memory capacity, a list of processors, and the number of cores. Many hosts or physical machines come together and form the data center. One or more VMs that include CPU, storage, and RAM are given for each host. In the data center architecture, 32-bit servers, XEN VMM, and Linux-based operating systems have been used.

The simulator supports the preparation of VMs in two levels, such as the host level and the VM level. The amount of total processing power of each core allocated to each VM is determined at the host level. A fixed amount of accessible processing power is allocated to the VM level’s unique application services (task units). VMs are hosted on the server, and they have access to the memory and processor of their owns host. A VM may be fully placed on a host or can be a part of a VM or migrate from a host to another host and run on it. The VM policy for allocating resources is specified in the cloudlet scheduler. In this system, there are 10 VMs; one of them is shown in Table 1. In this machine, the amount of RAM is 128 MB, the hard drive is 250 GB, and its used xenon architecture.

#### Cloudlet class

In the cloudlet modeling class, the types of tasks taken into the system are modeled. Before the run, each program in CloudSim will be specified by the size of the data and the volume of the processing. Also, researchers can rewrite this class and describe other types of tasks. The cloudlet is the same tasks or tasks that are supposed to be done in the cloud environment. In this paper, there are 10 cloudlets defined with different characteristics. Plus, the structure of the ants is in the form of a matrix. The number of hosts and VMs equals 10. We also set Max_Speed to 1 and Max_TTL to 5. Each ant is a task in the form of a matrix of 10∗2. Rows represent the number of the tasks, and columns represent which machine is in the executable mode. For example, when the matrix is set to [1, 2], it means that an ant number 1 (i.e., task number 1) runs on machine 2. Also, the amount of deposit pheromone per machine is initialized to 0.009. The pher-edge matrix is created in the form of 10∗10, and it stores the pheromone value of each machine. In the random movement of ants between machines, the ant’s path is stored based on the amount of pheromone in the form of a matrix to avoid returning to the same path and the same node; the structure of ant-path-matrix is in the form of 10∗10. In the ant-path-matrix, those paths that have the most pheromones are selected for the heaviest tasks.

#### Power class

This method defines a maximum power variable and defines a threshold (70%) for power consumption in the data center, using up to 70% of that capacity. This threshold is determined by the total power of the data center. But, it is not related to the power of the VMs; also, 70% is a random amount that is used to determine the actual amount of power, and that can be any other value. As a result, 70% is a reasonable amount for this purpose. In this paper, the processor, disk storage, power supply, memory, and cooling systems are used to determine the amount of consumed energy by computing nodes in data centers. According to recent studies, the energy consumption by servers is a roughly linear relation with the processor efficiency. Using DVFS limits the number of states that can be set for frequency and voltage. Therefore, other system components cannot use performance, scalability, and voltage. However, due to the growing number of modern processors, today’s modern servers have a large memory capacity consumed a lot.

### Dataset and simulation parameters

The requirements and goals of cloud service consumers and providers are usually different and opposed to each other. An SLA should be established between consumers and providers to provide a solution (Shojaiemehr, Rahmani & Qader, 2017). The definition of the used parameters in this paper is as follows.

- SLA differences: This parameter is one of the main parameters for system optimization. This parameter represents the level of agreement between the user and the server. Based on this parameter, whatever given service level to users is higher, SLA differences will decrease. This parameter is the difference between users’ total requests and the total number of requests in the system. This amount can be retrieved after the simulation by CloudSim because it simulates the real environment and considers all the parameters.
- Response time: It represents the total time needed to complete all the tasks, plus the time it waits and the time it takes to send and receive responses to the user.
- Energy: It represents the amount of consumed power to do tasks per machine; whatever this amount is less, resource management is well done, and there is no overhead on the system.

### Comparison

To evaluate the proposed method, 10 machines have been used to test the system. Firstly, the three benchmark methods are used to obtain the results. Secondly, the CSD_IACO is used to improve the system. To do this, the CloudSim library was used in the Netbeans environment. The parameters, such as energy consumption, SLA, and total time have been used to evaluate algorithms. By using four methods, the experiments have been performed. Note that the number of experiments was 10, and the number of tasks and machines was 10. The system’s SLA differences represent the number of unmet requests to the number of requests that have been met. The lower value of this parameter is better. If the difference between the agreements is less, it is preferable because both sides are close to a common agreement.

Table 2 describes the performance of the system. The CSD_IACO performs the better discovery performance. Besides, by providing a lower energy consumption, shorter response time, and lower SLA, the SSSD method surpasses the ACOD method. For example, compared to the ACOD, the CSD_IACO reduces energy consumption by 18.52%, response time by 18.96%, and SLA difference by 19.6%, on average. The CSD_IACO algorithm has a better load balancing than other approaches, which is one of the key reasons for its superiority. A reduction in pressure on a single node would benefit from a proper distribution of requests and load balancing between different nodes. As a result, proper load balancing has been accomplished. So, requests are responded to more quickly, and energy use is reduced. Cloud discovery with the CSD_IACO method further improves performance, e.g., decreases energy consumption by 14.29%, response time by 13.55%, and SLA by 10.2% compared to the SSSD scheme on average. So, the CSD_IACO method is an ACO spin-off that has many benefits such as good load balancing, better use of resources, and improving cloud discovery efficiency in general. We also see that the CSD_IACO method outperforms the FIPA-CD method to minimize energy usage by 10.7%, response time by 6.36%, and decreasing SLA by 3.54%. This result indicates the superiority of the CSD_IACO method.

**Table 2:** The amount of energy consumed, response time, and SLA differences by using the four algorithms in 10 times experiments.

Experiment	CSD_IACO			FIPA-CD			SSSD			ACOD		
	Energy (kWh)	Response time (second)	SLA	Energy (kWh)	Response time (second)	SLA	Energy (kWh)	Response time (second)	SLA	Energy (kWh)	Response time (second)	SLA
1	0.42	545	*545.62*	0.46	580	565.23	0.49	610	610.32	0.55	660	*660.57*
2	0.44	530	*526.52*	0.48	566	585.63	0.52	575	573.77	0.54	654	*590.36*
3	0.43	560	*535.62*	0.47	587	546.33	0.51	600	560.75	0.53	652	*723.96*
4	0.42	576	*490.63*	0.48	590	544.92	0.52	604	550.20	0.55	650	*610.36*
5	0.40	545	*461.23*	0.47	580	565.25	0.52	610	610.43	0.56	640	*636.21*
6	0.40	530	*540.96*	0.49	573	580.53	0.53	601	572.76	0.56	652	*658.38*
7	0.40.5	490	*509.36*	0.44	560	566.22	0.56	600	600.53	0.56	682	*602.36*
8	0.43	565	*473.32*	0.48	571	530.52	0.56	613	589.16	0.57	677	*618.23*
9	0.39	533	*525.12*	0.46	587	560.72	0.52	602	587.68	0.55	684	*653.29*
10	0.40	537	*540.87*	0.47	589	590.24	0.55	599	620.42	0.59	664	*671.25*

**Figure 5:**
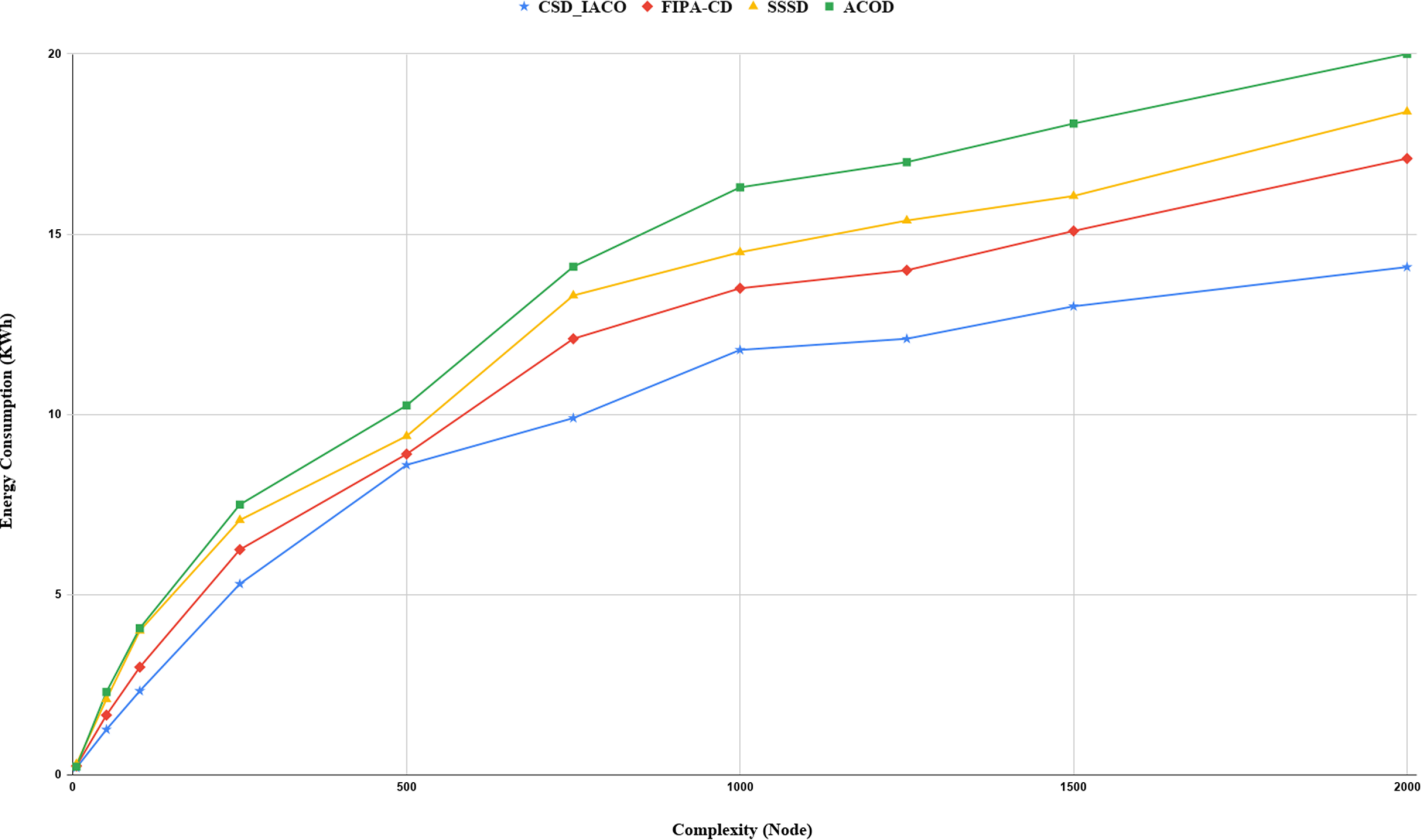
Energy consumption with increasing complexity.

**Figure 6:**
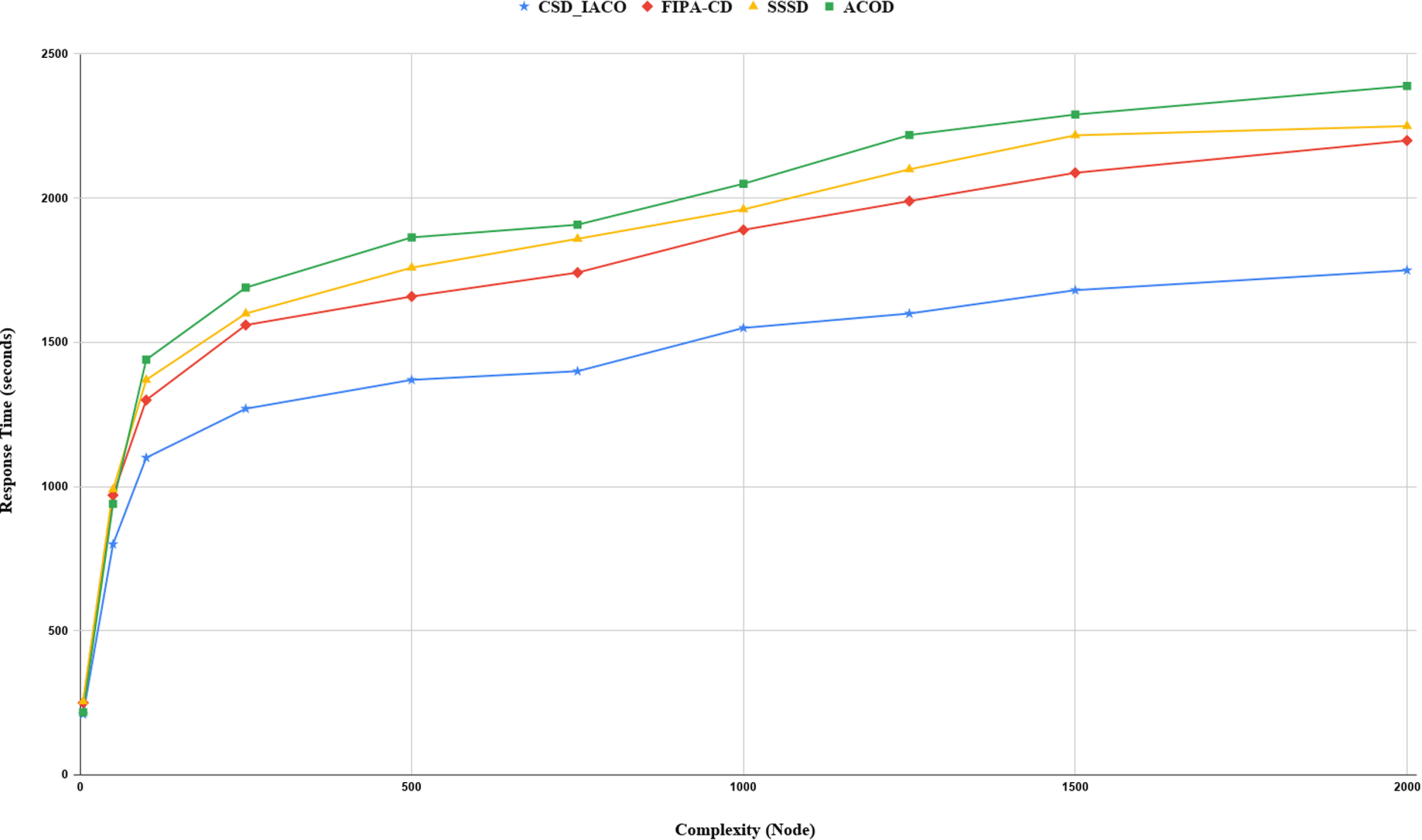
Response time with increasing complexity.

**Figure 7:**
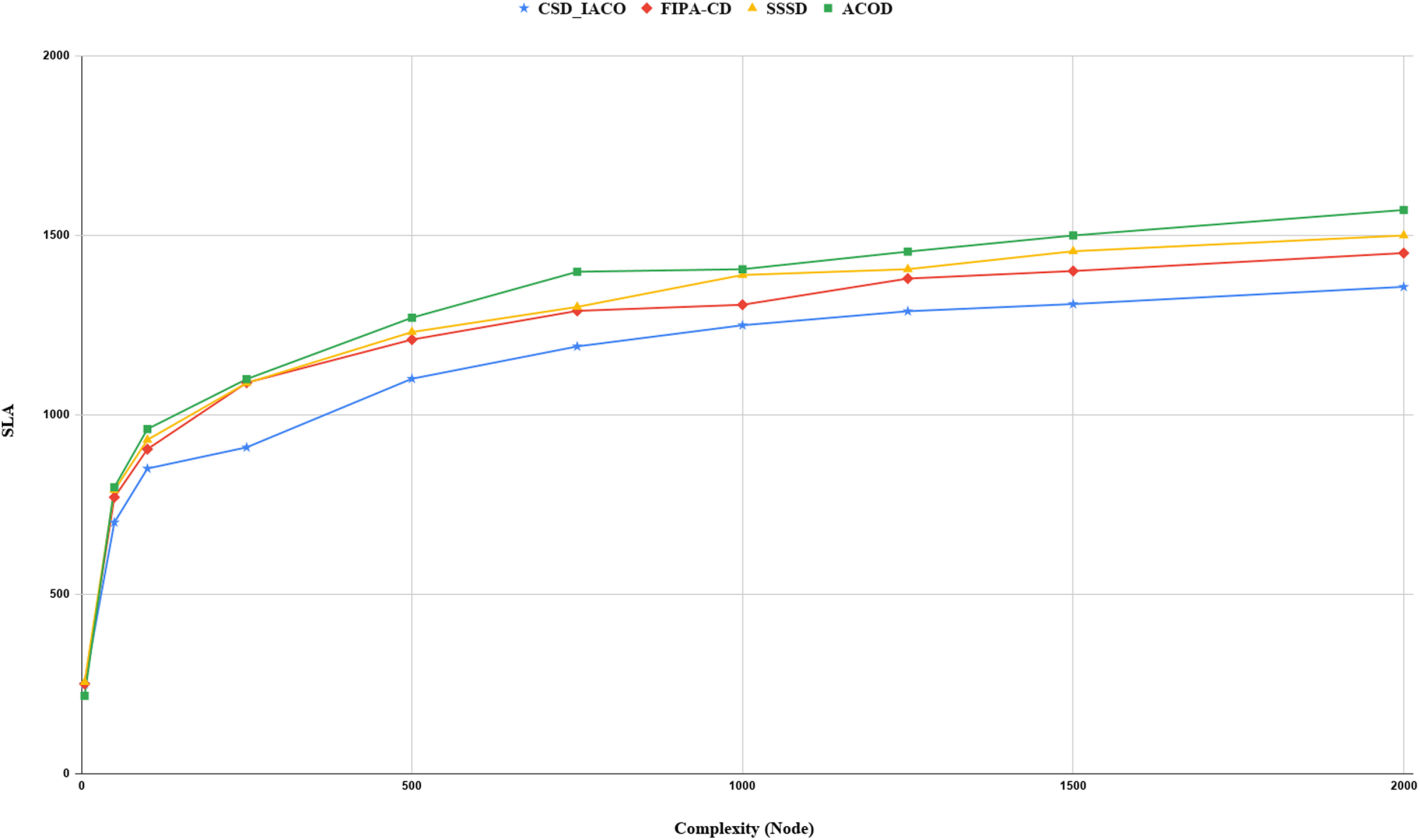
SLA values with increasing complexity.

We increased the complexity of the system to evaluate and analyze approaches by increasing the nodes. Figs. 5–7. demonstrated the relationship between increased system complexity and response time, energy consumption, and SLA. In cloud service discovery, the average discovery output is investigated during the condition of 750 nodes. Likewise, For example, Fig. 5. sets out the relationship between the increasing complexity and the system’s energy consumption. Obviously, with the growing number of nodes and tasks, the response time, SLA, and energy consumption of the system are rising. Also, as the number of nodes in the CSD_IACO method grows, the number of visited nodes grows as well, reducing response time and energy consumption in comparison to other approaches. For example, if the number of nodes increases 500 to 750, the CSD_IACO approach increases energy consumption, response time, and SLA by 15.12%, 4.19%, and 8.18%, respectively. The CSD_IACO also exceeds the SSSD with 16.56% lower consumption of energy, 18.69% lower response time, and 8.46% lower SLA, and exceeds the FIPA-CD method with 12.18% lower energy consumption, 18.63% lower response time, and 7.68% SLA. However, the average improvement percentage of all three parameters for CSD_IACO than other methods with raising the system’s complexity is shown in Table 3. One of the main reasons for the CSD_IACO method’s superiority is that there is a load balancing property in our proposed algorithm, decreasing traffic along the path and the burden on the nodes. Therefore, user requests are evenly distributed across all servers, decreasing energy consumption, reducing response time, and avoiding node bottlenecks.

**Table 3:** Average improvement percentage of all three parameters.

Parameter	Average percentage improvement compared to FIPA-CD (%)	Average percentage improvement compared to SSSD (%)	Average percentage improvement compared to ACOD (%)
Energy consumption	13.3	16.7	19.1
Response time	16.4	19.4	21
SLA	6.6	9.1	12.2

## Conclusion, Limitations, and Future Work

In the CSD_IACO method, based on the IACO algorithm’s steps, ants are assigned to each machine, and then the ant randomly began to move at each step. Three parameters were measured based on the mathematical model of the algorithm. In the end, each ant moves to the best machine to achieve the best service. Based on the results, the advantages of this method are high scalability, low response time, low energy consumption, better SLA differences, and high load balancing. Therefore, CSD_IACO will have high performance in managing a decentralized system. However, the major contributions in this article are (1) Suggesting a new decentralized discovery model for cloud service discovery (2) Propose a new mechanism for service discovery using the CSD_IACO method (3) Significant improvement in reducing response time, better SLA difference performance, and mitigating energy consumption.

Like any algorithm, the proposed method has some disadvantages, such as the following: Firstly, the parameter of the maximum number of steps, which means the step size of each ant that should be determined appropriately, and the correct determination of this number is very challenging. Secondly, the response time is still high, but it is suitable rather than the other three evaluated algorithms. The authors also assessed the CSD_IACO method to reduce energy consumption, response time, and SLA differences in cloud computing using the CloudSim simulator. Reducing energy consumption, the response time, and the SLA differences in the system’s whole are the proposed method’s efficiencies. The CSD_IACO algorithm’s efficiency is proved by performing the experiments based on the three benchmarks. By comparing the experiments’ results between these four methods, acceptable results are obtained for the CSD_IACO method. However, the main advantage of the approach is a shorter response time than others. Also, the algorithm leads to a load balance of the network, thus reduces energy consumption.

As suggested for future work, we can improve the proposed algorithm by combining it with other algorithms and using different evolutionary approaches. Of course, we can use another discrete meta-heuristic algorithm to compare the results better.

## Supplemental Information

10.7717/peerj-cs.539/supp-1 Supplemental Information 1 The simulation file.

10.7717/peerj-cs.539/supp-2 Supplemental Information 2 Simulation guidance.

## Additional Information and Declarations

### Data Availability

The following information was supplied regarding data availability: The code and simulation are available in the Supplemental File.
